# Reciprocity: A Predictor of Mental Health and Continuity in Elderly People's Relationships? A Review

**DOI:** 10.1155/2010/340161

**Published:** 2010-09-14

**Authors:** Live Fyrand

**Affiliations:** Department of Research, Diakonhjemmet University College, P.O. Box 184, Vinderen, 0319 Oslo, Norway

## Abstract

Many studies have demonstrated that social relationships confer mental health benefits. This paper aims to identify whether and how *reciprocity* in social relationships predicts or is associated with mental health benefits as well as with continuity in elderly people's social relationships. The studies reviewed in this paper show that, among elders, being in a balanced or underbenefited reciprocal position predicts better mental health and life quality than being in an overbenefited position. Throughout the course of life, reciprocity evens out present and earlier reciprocal imbalances, securing continuity in close relationships—particularly between spouses and between elderly parents and adult children. In friendships, securing continuity seems to be based on the maintenance of independence based on balanced reciprocal relations, making these relationships more vulnerable. Due to the problems of conceptualization and measurement in the reviewed studies, one should be cautious in stating a final conclusion that the reciprocity norm has a universal positive effect on mental health and continuity in elderly people's relationships.

## 1. Introduction

Paradoxically, while a huge number of empirical studies have investigated the mental health benefits of being connected to social relations and social groups [1], very few studies have investigated the basics for social relationships. Reciprocity is regarded as a basis mechanism that creates stable social relationships in a person's life [2]. It has been described generally as the process of “give-and-take”—the degree of balance in social exchange between people. Nevertheless, few studies of social relations among elderly people have been undertaken in which reciprocity has been a key variable.

In addition to the changes that aging brings to people's health in general and their way of functioning in daily life, aging people usually need increased help from others due to increased age-related health problems. This may imply that they—sooner or later—will be in a disadvantaged position in the “exchange market”—most frequently being in a receiving position due to increased need for help combined with less power and prestige [3]. According to the exchange theory, being more dependent on others may cause unbalanced relationships, with associated mental distress and discontinuity as possible negative consequences. Therefore, elderly people may be more exposed to mental distress and social isolation than younger people who are less dependent on others. These age-related changes may in turn influence elderly people's social interactions, creating a need to understand possible mechanisms of these relational challenges. A better understanding of such mechanisms may help us prevent relational discontinuity and negative mental health consequences of the aging process in elderly people.

This paper examines whether and how reciprocity can predict mental health benefits or continuity in elderly people's social relations. This may represent an expansion of knowledge and tools based on a bio-psycho-social perspective for professionals working with elderly persons.

### 1.1. Important Theoretical Concepts

There seems to be four important concepts in the reciprocity field of theory which are interconnected in the description of important presupposition for social interaction: *reciprocity, exchange, equity, and indebtedness*.

The concepts of “*exchange*” and “*reciprocity*” are closely related and often used as synonyms. However, while exchange can be defined as “*the action of, or act of, reciprocal giving, and receiving,”* reciprocity is defined as a “*mutual action, influence, giving, and taking”* [4]. Exchange of resources is by social psychologists seen as the social events most relevant to relationship formation and maintenance. Gouldner [2] regarded the norm of reciprocity as the basic assumption for social exchange interactions, viewing this norm as universal as the incest taboo and a “starting mechanism” in people's motivation to initiate contact and interaction on a utilitarian basis. The reciprocity norm determines the value and level of the content in the exchange process, implicitly setting the equivalence standard (i.e., stipulating the repayment to be “roughly equivalent” to the value of what has been received). This is meant to prevent reciprocal imbalance or inequality which further may create relational instability between the exchange partners.

While the reciprocity concept is basic describing the degree of balance of the transactions in the social exchange, is *equity *a more subjective assessment. The degree of reciprocity can be characterized as equitable in its fairness and justification based on the persons' values and norms. According to the equity theory is fairness in relationships with others depending on the observer's perceptions and assessments of the value and relevance of the participants' inputs and outcomes [5], being “in the eye of the beholder”. Thus, it is viewed as a basic value and not as a social norm. When the amount of support received is more or less than that provided, we talk about being in an imbalanced and undesirable status [6].

There seem to be two kinds of imbalanced status: (1) an overbenefited status, meaning that a person is receiving more than giving which can be related to a feeling of indebtedness, guilt, and shame, and (2) an underbenefited status, providing more support than one receives which may lead to feelings of resentment, burden, and dissatisfaction [7]. While both equity and exchange theories suggest that individuals will be dissatisfied in underbenefited relationships (less received than provided), equity theory states that individuals also will experience overbenefited relationships as unsatisfactory, which is in contrast to exchange theory [8].

According to equity theory may lack of repayment for the help received place the receiver in a psychological state of indebtedness to the provider of help causing a threat to the individuals independence. This may lead to psychological distress for the indebted receiver [9]. Thus, equity theory assumes that receiving support/help may alter the subjective feeling whether there is an equitable balance of exchanges within a relationship. Empirical studies show that the greater the inequity between the partners, the more distress, and the harder they will try to restore equity [9, 10]. Resisting support may also be understood as a way older people maintain their ego identity [11].

Giving, receiving, and repaying are crucial parts in three different phases in a reciprocal exchange process [12]. In unequal relationships (where outcome is unequal rewards minus costs), a feeling of *indebtedness* toward the donor (provider of help) may develop. While the provider of a gift most often will feel independent toward the receiver, will the receiver on the contrary often experience a dependence of the provider due to the problem of indebtedness toward the donor. Refusing gifts or the offer of help may therefore prevent the potential receivers' feelings of inequality and indebtedness towards the donor (i.e., chronic patients experiencing increased need for provision of help as the disease progresses often refuse to accept help). Indebtedness, with no immediate possibilities to be reduced, inhibits a person's help seeking [13]. Considerations of costs and of immediate opportunities for the receiver's repayment of his/her obligations seemed to be less important in close relationships where inequities will “even out” in the long run. Inequities will constantly occur in close relationships compared to more distant relationships.

Even if indebtedness derives from inequity, it differs in the way that equity theory views recipients as being motivated to restore equity to the donor-recipient relationship, whereas indebtedness theory views recipients as motivated to reduce their indebtedness to the donor. Gouldner [2] describes this by stating that “the shadow of indebtedness” will fall over the time between the provision of a benefit and the time for repayment. Greenberg [14] expresses a warning both to the “would-be donors” and to the “would-be recipients” about the attendant risks for both parties following the exchange process in a relationship. The warning should especially go to the would-be recipient, having responsibility for the problem and the help needed, thereby implying risks for an increase of indebtedness to the donor. *“In our eagerness to find ways to help needy populations (such as the elderly and the handicapped), perhaps we have too often overlooked one of the most genuinely rewarding and mutually satisfying arrangements- encouraging the “needy” to give useful help as well as to receive it*” [13]. Democritus (fourth century B.C.) offered the following advice: “*Accept favors in the foreknowledge that you will have to give a greater return for them”* [14].

The word reciprocity can also mean “inversely related”, differentiating between (1) *symmetrical* (giving the same contributions to each other) and (2) *complementary reciprocities* (the contributions are not equivalent but compensatory or complementary) [15]. Even if reciprocity for the most has family and kinship as its basis [16] characterized by complementary reciprocity, is symmetrical reciprocity in particular related to friendship relationships. *Balanced reciprocity* (which can be basic in all relationships) is by definition more specific and short-term exchanges used especially in relation to more distant relationships (remote kin, neighbors, etc.) [17]. However, according to Sahlins [18] is balanced reciprocity basic in all relationships referring to the exchanges where there is a balance in which an equivalent of the thing received is returned within a finite-time period. Sahlins views balanced reciprocity as a third point between a continuum with positive and negative reciprocity at the two ends. This may be seen as the continuum on which all of us move in a psychological and social process toward our relationships whether it is close family, friends, or colleagues due to our age, gender and the life-challenges we are confronting during our life time. Neither our relationships nor we are static, but dynamic developmental processes from the earliest childhood until our death.

While symmetrical and complementary reciprocity are interactions between the same people who exchange different kinds of transactions, can *generalized*, *waived, constructed * [19, 20], and *stepwise reciprocity* [21] be directed towards other people than to the providers of benefits.

While *generalized* reciprocity is described as an altruistic characteristic of networks where given support is not expected to be returned in the same proportion and from the same people, was *constructed *reciprocity mainly used where the caregiver had had a long-lasting relationship to the care recipient. This type of reciprocity form was mainly used in relation to confused and ambiguous persons where the caregiver most often had to interpret the recipient's nonverbal communication about their needs. *Waived* reciprocity occurred when expectations of immediate reciprocity were relinquished. The caregiver had an open-ended time period in their reciprocity assessment and some caregivers did not expect reciprocity at all [19, 20].

In *stepwise reciprocity* does the assistance from the caregiver go from the recipients to some new receiver and not back to the original provider of the support received [21]. The reciprocity will then have a kind of stepwise form where help is provided in the first step from A to B, where B in the next step covers his/her need to reciprocate for the help received from A further to person C. “Pay-it-forward” is a metaphor which may describe this kind of reciprocity.

### 1.2. Search Strategy and Selection Criteria

The identified studies focused mainly on the relationship between *reciprocity and continuity* and between *reciprocity and mental health* in the social relationships of older people. The empirical studies accessed for this paper were identified both by* references in published articles* and by *a literature search* using the following key words individually and in combination: “elderly”, “social exchange”, “reciprocity”, “equity”, “indebtedness”, “mental health”, and “continuity of social relations”. The keywords for our literature search were located in the abstracts of the articles. The following OVID databases were searched: Medline, PsychINFO, and ISI Web of Science. While ISI Web of Science is an interdisciplinary database and Medline covers the general medical field, PsychINFO specifically covers the field of mental health. Both cross-sectional and longitudinal studies published in English, on elderly people aged 50 years or more, were included in this paper. All the studies identified in our literature search were included without any quality assessment procedure performed in the selection of the studies. This was because our literature search revealed *a lack of research and published studies in this particular field of interest. *The literature search had no limitations on publishing years. Twenty studies were identified. Twelve of these investigated the relationship between reciprocity and continuity in elders' social relations, and eight studies focused on the relationship between reciprocity and mental health. Empirical studies on relationships between reciprocity and mental health between professionals and elderly patients have not been included due to the difference in topic.

## 2. Reciprocity and Relational Continuity

### 2.1. Life-Course Reciprocity Evens Out Present and Prior Reciprocal Imbalances

Different kinds of social support (emotional and instrumental support, social companionship) are exchanged in different ways according to the particular relationship. For example, social support types differ between spouses, children, and friends [22]. This may imply that the reciprocity norm is practiced in different ways according to multifactor situations and therefore must be taken into account when seeking to understand the relationship of reciprocity to the continuity of elderly adults' social relationships.

Relational continuity seems to be a basic need in the lives of people and families, based on a universal and cross-cultural human expectation [23]. Stable social relationships seems to be important due to the impact of social relationships on an individual's somatic and mental health [1, 24].

Eight studies were found focusing on the relation between reciprocity and continuity in elders' social relationships [7, 25–31]. 

The studies of Silverstein et al. [25] and Becker et al. [26] focused on intergenerational reciprocity between adult children and elderly parents. Silverstein et al. [25] examined why adult children provided support to their elderly parents over a 27-year period of time comprising 501 children; 416 child-mother and 317 child-father relationships living in California, USA. Becker et al. [26] investigated relationships between adult children and elderly parents in four ethnic groups in the United States. Five in-depth interviews of 270 respondents older than 50 years of age were conducted over a 5-year period. This study focused particularly on how the culture-specific conceptions of mutual assistance reflected the nature of social exchange and its role in creating continuity in family relationships.

Intergenerational reciprocity was found to be assumed for continuity of intergenerational relationships [25, 26]. Adult children's earlier family experiences, particularly of emotional and social activities with their parents during their growing-up period, proved to be investments in reciprocal activities and support from the same children toward their elderly and retired parents when age-related needs for help, support, and company emerged among elders [25]. Therefore, children's motivation to provide social support to their older parents seems to be rooted in earlier childhood experiences with their parents. However, Becker et al. [26] found that when traditional cultural values were not reciprocated through deferential behavior by children, a breakdown in communication occurred. A breakdown of mutual assistance across generations was also found if there was a shift in the extended family away from cultural values supporting traditional family patterns.

In two longitudinal studies, Klein Ikkink and van Tilburg investigated whether reciprocity predicted continuity in elderly people's social relationships and prevented termination of elders' ties in 408 [27] and 2057 [28] elderly Dutch men and women living independently (mean age, 68 years). The respondents were drawn from the population registers of 11 municipalities representing differences in culture, religion, urbanization, and aging in the Netherlands. Eight types of relationships were included in their studies.

The studies of Klein Ikkink and van Tilburg demonstrated that relationships among close kin are most likely to be continued, whereas more peripheral relationships are more vulnerable to termination [27, 28]. Over a year, unbalanced relationships became more balanced; the initially balanced ties still remained balanced, as well. However, even if being overbenefited by their close kin was experienced as an uncomfortable situation, it did not represent a threat to the continuity of the relationship, probably due to the norm that reciprocity cannot be fully applied between elderly people and their children [27]. Being overbenefited of instrumental support from family members—having a reciprocal imbalance in the present—seems to restore and even out earlier imbalances between close family members that may have been caused by past overbenefiting of instrumental support toward family. This practical help provided by close kin relationships made elderly people more independent toward their friends.

Antonucci and Akiyama [29] as well as Ingersoll-Dayton and Antonucci [7], in a cross-sectional study, investigated the perceptions of reciprocal and nonreciprocal emotional and instrumental (sick care) support in relation to spouses, children, and friends in a sample of 718 middle-aged and elderly adults ≥50 y of age in the United States, focusing on the reciprocal lifespan perspective in three different agegroups. According to the relationship type, the spousal relations were found to be most reciprocal compared with other relational types [29]. In addition the “older elders'” seemed to feel more comfortable being overbenefited by receiving instrumental support from children, taking into account a life-course perspective with previous life periods when the older persons provided family members with more support than they received. “*A sense of life-course reciprocity is probably more difficult to adopt with friends or others who are known for shorter periods of time”* [29].

While life-course reciprocity seems to be more difficult to practice in relation to friends or others who are known for shorter periods of time, spouses and children adopt a perspective of life-course reciprocity [7]. Even if the relationships change due to the aging of the persons involved and their independent life experiences, parents and children usually remain in each other's relational convoys [29]. With the exception of spouses, both relationships with children and friends were perceived as somewhat less reciprocal. Overall, the equity theory perspective (the need for balanced reciprocal relationships) was more important for adults in relation to their children than their spouses, where greater imbalance was tolerated.

Lewittes [30] examined the relation between reciprocity and the duration of elders' friendships in her cross-sectional study of friendship patterns and interaction skills among 169 white and black elderly women, older than 65 years of age, living in the suburban and urban neighborhoods of Long Island and the greater New York City area. This study provides interesting information about presuppositions for the development of reciprocity in friendship relationships for elderly women. Similarities in ethnic membership, social class, and age all contributed to the likelihood of a reciprocal pattern. Reciprocal relationships seemed to include both similar and complementary exchanges. Elderly women seemed to find it more acceptable to give than to receive from friends causing a reluctance to seek help or to maintain relationships when they were on the receiving end (i.e., being in an underbenefited position). A value and norm of independence prevails, implying that it is the family that the elderly woman should turn to when help is needed.

This is partly supported by the results of a longitudinal study using participant observation and interviews, conducted by James et al. [31], of 70 people over 60 years of age living in three coastal rural areas in Western Ireland. The study comprised four waves from 1978 to 1982, showing that exchanges with more remote kin and neighbors seem to be more specific and short term (i.e., balanced reciprocity) in comparison to parent/child relationships, where expectations of return by parents from children are long term and without conscious thought of present return (i.e., generalized reciprocity). Therefore, parent/child relationships may tolerate greater present imbalance than relationships that are more likely to feature constructed or generalized reciprocity, such as relationships with friends, neighbors, and more distant relations.

The importance of having a lifespan perspective to understand the relationship between reciprocity and continuity of relations in an older-adults network, were shown in these studies. The life-course reciprocity was mainly practiced in relation to spouses and children, where former underbenefitings of support were evened out in older age. Friends however, seemed not to be included in a life-course reciprocity process. It seems easier to give than to receive from friends according to a value of independence in friendship relationships compared to family ties. Older adults tie duration to friends seemed thereby to be more vulnerable due to present nonreciprocal exchange caused by the lack of lifecourse reciprocity.

### 2.2. The Reciprocity Norm Is Practiced in Many Different Ways

While three studies give important information about reciprocity as a prerequisite for the continuity of social relationships between caregivers and elderly patients [20, 32, 33], one study has investigated the relation between reciprocity and time duration with different subgroups before and after entering residential care homes [34].

In a two-year followup study of 53 (22 male/31 female) spouse caregivers (mean age was 68 years for caregivers and 71 years for patients), de Vugt et al. [32] investigated relationship problems due to behavioral issues of care recipients with dementia, and mental health problems of spouse caregivers caring for patients with Alzheimer's and Parkinson's diseases. The study was part of the Maastricht Study of Behaviour in Dementia in the Netherlands. Apathy and withdrawal were the most disruptive patient behavioral problems, and hindered the caregivers' ability to share their thoughts and feelings with patients, negatively affecting the reciprocity of the relationship between the caregiver and the care recipient regardless of the sex of the caregiver.

A cross-sectional study by Hooker et al. [33] focused on respondents living in upstate New York; respondents were 175 spouse caregivers for patients with Alzheimer's disease (*N* = 88: m 36/fm 52; caregivers' mean age: 71 y) and Parkinson's disease (*N* = 87: m 32/fm 55; caregivers' age: 67 y). Although the results from this study mainly support the conclusions of de Vugt et al. [32], they differ with respect to the caregiver's gender. Among the Alzheimer's care group, female caregivers (wives) reported worse mental health outcomes than the male caregivers (husbands), due to loss of reciprocity as a result of cognitive deterioration in care recipients. On this basis, the researchers concluded that loss of reciprocity in marital relationships may affect women more negatively than men:

“...to the extent that women are more likely than men to fuse marital satisfaction with general well-being, they will be at a disadvantage in the dementia care giving scenario because of the loss of reciprocity. This phenomenon may be more common among women, but is certainly not specific to them.” [33].

Neufeld and Harrison [20] reveal interesting findings in their longitudinal study of 22 male caregivers—18 of them ≥60 years of age—providing care to recipients (wives ≥60 years of age) with cognitive impairment caused primarily by Alzheimer's disease, living in Canada. None of the men described any reciprocity in their current relationship with the care recipient. The present lack of reciprocity in the relationships between caregivers and care recipients was found to be compensated in different ways that prevented discontinuity in these relationships. Three variations of indirect reciprocity that compensated for lack of present reciprocity were identified: constructed, waived, and generalized. Constructed reciprocity (attendance to nonverbal behavior) was used by the caregivers who felt they were committed to an ongoing relationship with their wives, based on positive, long-term relationships with them prior to the onset of cognitive impairment. The lengths of their marriages varied from 27 to 60 years, and they viewed the difficulties they faced through the lens of these relationships. However, waived and generalized reciprocity were based on a view of reciprocity as a moral norm, which may be either suspended (waived) or fulfilled by contributions to a third party (generalized). The men who gave care by obligation described their feelings in this situation as burdened, stressed, angry, lonely, and frustrated.

Bear [34] investigated the relationship between social network characteristics (including reciprocity) and tie duration among the 75 of the closest family and friends of 81 new elderly RCH residents in central Florida, USA (≥60 years of age), at RCH-entry and six months later in a face-to-face interview. Reciprocity proved to have an effect on the elderly people's tie duration with their primary ties after entry into a residential care home. Although reciprocity directly affected the frequency of visits with their relatives and both visits and speaking contact (via telephone) with their closest family and friends, present reciprocity showed no general effect on tie duration with friends. Relatives and friends continued to help the residents both directly and indirectly (generalized reciprocity) by helping the closest network members to maintain their direct support of the residents. Indirect help was explained by the fact that friends were repaying the residents for past services by helping the closest ties to maintain their contact with the residents.

## 3. Reciprocity and Mental Health

### 3.1. A Balanced or Underbenefited Position Predicts Better Mental Health

In addition to the twelve previously reviewed studies on the relation between reciprocity and continuity in elders' social relations, we identified seven studies that focused on the relation between reciprocity and mental health.

The effort-reward imbalance model—which was tested in a cross-sectional study by von dem Knesebeck and Siegrist [35] on marital, parental, and unspecific relational roles—investigated a potential imbalance between effort spent (“high cost”) and rewards received (“low gain”) in the subjects' social exchanges. The study comprised 1290 noninstitutionalized elderly men and women ≥60 years of age: 682 in Germany (mean age, 70.8) and 608 in the United States (mean age, 72.3). Study results revealed consistent associations of nonreciprocal social exchange with depressive symptoms for both genders in both samples. The risk of depressive symptoms was about twice as high among elderly men and women who reported nonreciprocity in their social exchanges compared to subjects reporting reciprocal social exchange.

Furthermore, studies on the relationship between reciprocal social exchange for health and well-being tested the associations of different types of social activities (paid work, caring, and volunteering) and well-being [36], and between social productivity (voluntary or charity work, caring for a sick or disabled adult, and provision of help to family, friends, or neighbors) and well-being [37]. According to Siegrist et al. [38], socially-productive activities are based on the social norm of reciprocity, “…*in which the effort of doing the activity is made in anticipation of an equivalent reward that reflects the value of the effort involved*” [36]. Data from a cross-sectional wave in 2004 of the English Longitudinal Study of Ageing (ELSA), of 5384 participants at post-state pension age (≥60 years for women and ≥65 years for men), were analyzed to examine whether participation in social activities (i.e., caring for another person) was associated with higher levels of well-being (i.e., depression), and explained by “the reciprocal nature of these activities” [36]. The study showed that reciprocal exchange had a negative association with the degree of depression regarding the activity of caring [36]. The cross-sectional study of Wahrendorf et al. [37], was also investigating the relationship between social activity (defined as social productivity, i.e., caring for a sick or disabled person and provision of help to family) and well-being (i.e., depression), focusing on the quality of the activity based on the notion of exchange reciprocity. The study was accomplished on 22 000 participants, ≥50 and from ten European countries—using data from the SHARE study (“Survey of Health Aging and Retirement in Europe”). The study uncovered that reciprocal activity was associated with lower scores on depression both for caring and informal help. Thus, all three studies [36–38] are lending “…*support to Siegrist's observations on the importance of reciprocal exchange in social relations for health and wellbeing in later life*” [36].

Liang et al.'s [39] cross-sectional study of a national probability sample of 1103 elderly individuals >65 years of age living in different counties in the United States, Roberto and Scott's [40] cross-sectional study of 110 noninstitutionalized white elderly people >65 years of age living in a southwestern city in the United States, and Rook's [41] cross-sectional study of 120 elderly widowed women (mean age, 72 years; range, 60 to 89 years) recruited from four senior centers in Los Angeles, California, demonstrated that underbenefited elderly persons had a decrease in psychological distress and had fewer feelings of loneliness, compared to overbenefited elderly persons who experienced the reverse situation. Nevertheless, overbenefited elderly respondents in the study by Roberto and Scott [40] reported more anger than underbenefited elderly respondents. This was explained by a substantial need for help and support and an inability to reciprocate. This type of situation may undermine elderly adults' sense of independence and self-worth due to a change in power in the elders' disfavor, compared with previous years [40]. Thus, these studies [39–41] revealed that elderly persons who were overbenefited or underbenefited were lonelier, experiencing more mental distress when compared to elderly persons whose relationships were reciprocal. 

However, psychological restoration of the feeling of inequality may be due to the elderly person's “equity potential” (i.e., personal and social competence, health, income [pensions], and available support network) [42]. In a cross-sectional study of 111 Japanese-American elderly persons (mean age, 74 years; range, 60 to 90 years) in New York City, Nemoto found that the elderly people who had strong reciprocity norms and were overbenefited had less life satisfaction. This differential in life satisfaction was due to what he called a “reciprocity-burden” to reciprocate help provided earlier in life, despite the impossibility of accomplishing the reciprocity norm due to age-related life challenges. In contrast, the overbenefited elderly who were unable to return the help provided and who had a weak reciprocity norm reported higher life satisfaction and showed fewer signs of aging.

### 3.2. Same Pattern Identified in Studies of Other Age Groups

Due to the findings in the previously reviewed studies on elderly people, we want to give a small amount of attention in the end of this paper to seven cross-sectional studies carried out with three populations of nonelderly participants: students [6, 43, 44], middle-aged people [45–47]_, _and parents to children with cancer [21]. These studies uncovered more or less the same pattern regarding the relationship between reciprocity and mental health, independent of the difference in the informants' life situations. Lack of reciprocity was generally associated with negative effects and poor mental health. Underbenefited as well as overbenefited students were confronted with negative feelings toward their partners, including feelings of dissatisfaction (underbenefited students) and indebtedness (overbenefited students). Feelings caused by reciprocal imbalance included feelings of indebtedness, dependency, incompetence to cope with life challenges, shame, decreased self-worth, and anger and seemed to have a negative influence on people's health.

### 3.3. The Importance of Maintaining Independence

Wentowski's study [17] can be viewed as a “summing-up” study of the previous studies, identifying as it does a general pattern of the relationship between reciprocity and aged people. Wentowski conducted a two-year anthropological investigation to obtain an insider's perspective on the interaction of support and exchange transactions within a network of 50 elderly people (mean age, 71 years; range, 55–83 years) living in the urban American South. Independence was the key concern for all the elderly people in this study. Regardless of which strategy they used predominantly, they all shared a reluctance to accept “charity”. For most people, independence is “interdependence”, accepting support from the network into which one has invested over a lifetime, and thereby practicing the reciprocity norm. Whatever their perception of independence, these people are using the same cultural system of meaning that surrounds the significance of reciprocity to create personal responses to the challenges of old age.

Therefore, it seems to be important to maintain independence in a variety of cultures. Against this backdrop, James et al. [31] viewed the importance of pensions for elderly people in the following way:

One of the values of pensions as a primary power resource is that they represent a stable source of income to maintain reciprocity. After land or a house is given away, reciprocal obligations will decline and dependence may increase but checks continue to be received [31].

The studies reviewed are shown in Table 1.

## 4. Conclusions and Applications

### 4.1. Conclusions

The aim of this paper was to identify whether and how reciprocity can predict health benefits as well as continuity in elderly people's social relations, to increase the awareness of reciprocity as a basic mechanism of health and social relations for elders in health professional's work with elderly people. 

Although the cross-sectional design of some of the reviewed studies demands that we be cautious in interpreting them, they all seemed to reveal that the degree of imbalance in social relations has significant value. Reciprocal imbalance seemed to have a potential to cause mental distress as well as both dependency and indebtedness, leading to a decrease in people's ability to cope with life challenges. The studies reviewed have revealed that both overbenefited and underbenefited women and men were lonelier, experienced more mental distress, and were less satisfied with their marital relationships than those whose relationships were reciprocal. Nevertheless, underbenefited elderly people seem to have less mental distress and fewer feelings of loneliness than overbenefited elders.

Furthermore, this paper reveals the importance of the lifespan perspective to understanding the relationship between reciprocity and continuity of relations in an elderly adult's network. Life-course reciprocity has mainly been practiced in the context of spousal relationships and parent/child relationships, where former underbenefiting of support was balanced out in older age. Friend relationships appeared to be excluded from the life-course reciprocity process. The norm of the maintenance of independence towards friends relies on present balanced reciprocity, making these relationships more vulnerable to changes in reciprocity. In a 10-year followup study of 1477 persons aged 70 years or more, Giles et al. [48] found that *“…greater networks with friends were protective against mortality in the 10 year follow up period” *[48], underscoring the importance of maintaining and developing friendships.

The caregiver's role is significant from a clinical point of view. Most caregiver respondents viewed reciprocity as a moral norm that could be suspended or fulfilled by contribution to a third part, compensating for lack of reciprocity in their present relationships with care recipients. Even so, most of them felt burdened, stressed, angry, lonely, and frustrated due to not living in a close reciprocal relationship. Female caregivers seemed to be more exposed to mental health problems than male caregivers due to an association between lack of reciprocity and marital satisfaction for women. Family caregivers were unlikely to withdraw from the relationship with the care recipient even if chronic conditions disrupted care recipients' cognitive functions, causing a lack of balanced reciprocal relationships.

An interesting finding was that the cultural reciprocity norm may regulate the exchange process in social interactions, thereby decreasing possible negative consequences for elders' life satisfaction and psychological well-being. While strong reciprocity norms may increase the feelings of aging and dissatisfaction in elderly people's lives, weak reciprocity norms may decrease these feelings. Elderly persons with a weak reciprocity norm—whether individual, cultural, or both—seem to be less indebted to the caregiver. According to Jou and Fukada [6]—who studied Japanese university students—reciprocal imbalance may cause even worse health consequences in Japan than in Western societies, due to the cultural importance of the reciprocity norm that maintains the value of harmony, compared to the value of independence in Western societies.

The interpretation of the results of the studies reviewed in this paper is restricted by some limitations. 

First, existing research reviewed in this paper is limited by both problems of conceptualization and measurement. The concept of social support, caring, and helping others and the type/role of relationship between the exchanging actors differs between the studies reviewed. They also vary a lot according to the research design and the measurement of the degree of reciprocity/balance in the relationships, the provision and receiving process in the exchange process [7, 29].

Second, some of the studies reviewed have taken a very short-term focus on issues of reciprocity and exchange, despite probable expectations that very close relationships are long-term phenomena. Adopting such a perspective may be insufficient to identify the extent of supportive exchanges across the lifespan—in particular of close relationships [8].

Third, the studies in this paper seem to reveal that reciprocity *may* have a significant impact on the continuity in people's relationships and on their mental health. However, the reviewed studies on reciprocity and mental health have a cross-sectional design which must be taken into account regarding the conclusions. It may be likely that health causes changes in reciprocity (i.e., a balanced, overbenefited or underbenefited position), in addition to reciprocal imbalances causing health problems. Longitudinal studies investigating causal hypotheses are therefore recommended to identify the cause-effect relationship of reciprocity to mental health.

In addition, the relatively few studies carried out regarding this paper's topics have also to be underscored.

Taken together, even if the results in the studies reviewed in this paper seem to support Gouldner's [2] statements that the reciprocity norm may be a utilitarian basis for relational stability and for a person's mental health, one should be cautious in stating a final conclusion that the reciprocity norm has a universal positive effect on mental health and continuity in elderly people's relationships.

Future research should consider using longitudinal design—in the field of reciprocity and social exchange processes for older people with the aim to identify the cause-effect relationship between the topics measured. Furthermore, the problems of conceptualization and measurements variability in this field require a more unified definition of central topics (i.e., social support, caring, and help to elderly people), classifying the elders relationships in terms of their different functions in the social network [8]. This implies that global measures (i.e., “social support”) should be avoided due to the fact that they do not capture the meaning of exchange within specific relations.

Standardized measurements (quantitative as qualitative) of the multiple factors in the reciprocity process in social exchange (i.e., degree of reciprocity: a balanced or an over- and underbenefited relational position) should be considered to compare results with similar studies. Measuring the multiple factors in an exchange—process seems to be important—both regarding the continuity of a functional social network and the elders mental health, due to the results uncovered in this paper. The research in the field of social network and mental health shows that a functional social network prevents social isolation and thereby a decrease of the elders mental health [1]. On this background further studies in this field should consider *continuity*/*tie duration/longevity of social relations* and *mental health* (life satisfaction, quality of life, mental distress, i.e., depression) as dependent variables of interest.

### 4.2. Applications in Clinical Work

Aging implies a transitional period where most of us gradually experience a decrease in our health and daily functioning, resulting in an increased need for help from others. These age-related changes may in turn influence elderly people's social interactions, creating a need to understand possible mechanisms of these relational challenges. According to the studies reviewed here, being more dependent on others may cause unbalanced relationships that can result in mental distress and discontinuity in social relations.

Based on these findings, we will suggest that health professionals who work with elderly people incorporate the conclusions from the reviewed findings into their clinical work with the aim of maintaining elder's independence in order to prevent relational discontinuity and concomitant increases in the mental distress of elderly people. When professional helpers enter into an elder's life as professional agents they must keep in mind the challenge represented by “the helplessness-independence” paradox (i.e., increased helplessness is followed by an increased need for independence [49]. Therefore, despite elderly persons' increased need for help—the “twin needs” (the need to receive help as age-related diseases occur and the need to maintain independence) should be noticed, both in relation to professional assistants and their personal network.

The significance of balanced reciprocal relationships for elderly persons demonstrated in this paper should be integrated as part of professionals' competence, attitude, and help provided to elderly patients, as well as into counseling and cooperating between the professional helper and the elderly's significant others. The aim should be to counsel caregivers and/or other persons in an elderly patient's life (and particularly friends) [49] to acknowledge the importance of creating or maintaining reciprocal balanced relationships and to avoid bringing an elderly patient into an overbenefited position with respect to the helper, as much as possible. Professional helpers should encourage elders and their social relations to maintain and increase reciprocal relationships, which may influence the longevity of the elder's social network, mental health, and length of survival.

## Figures and Tables

**Table 1:** Studies covered in this paper.

Author's name	Year	Type of study	Nationality	Sample groups	Sample age (≥ or/M)	Size (*N*)	Type of social support	Measures of reciprocity	Outcome variable
Silverstein et al.	2002	Longitudinal (27 y) 1971–1997 Six waves Questionnaires	USA, California; middle and working classes	Elderly parents children Intergenerational	1971–1997: Children: 19 y–43 y Parents: 45 y–72 y	*N* = 501 children	Emotional support Practical support Informational support	Dichotomously scored (0: “not provided”; 1: “provided”) each indicator and summed them to create an additive scale ranging from 0 to 5 at each time period, which were statistically analyzed.	Continuity

Becker et al.	2003	Longitudinal (5 y) Five waves Open-ended and semi-structured interviews	USA—four ethnic groups	Elders and their children/family Intergenerational	≥50 y	*N* = 270 elderly	“What kinds of support or assistance do elders provide to family members and vice versa?”	Comparison of coded answers of provision and receiving support	Continuity

Klein Ikkink et al.	1998	Longitudinal (3 y) 1992–1995 Two waves Mailed questionnaires	The Netherlands—Dutch ethnic group	The elders. Family subgroups, friends, neighbors, and acquaintances	≥55 y M: 68 y	*N* = 408	Instrumental support	Reciprocity variables were constructed by subtracting the support received by the support given. A negative score indicated “overbenefiting”; 0 meant “balance”; a positive score indicated “underbenefiting”.	Continuity

Klein Ikkink et al.	1999	Longitudinal (3 y) 1992–1995 Three waves Face-to-face interviews	The Netherlands—Dutch ethnic group	The elders. Family subgroups, friends, neighbors, and acquaintances	≥55 y M: 68 y	*N* = 2057	Emotional support Instrumental support	Reciprocity variables were constructed by subtracting the support received by the support given. A negative score indicated “overbenefiting”; 0 meant “balance”; a positive score indicated “underbenefiting”.	Continuity

Antonucci et al.	1987	Cross-sectional Personal interview	USA	Elders All members of a sampled household 70 y and older were interviewed	≥50 y (Range 50–95)	*N* = 718	Network structure (i.e., network size) and functional support to the ten most important network members (NM) regarding confiding, respect, caring, talking about health.	Respondents (NM) were asked to identify each network member from whom they received each type of support as well as whom they provided each type of support. Functional support where NM provided support was coded as 1. NM who did not provide support to the focal person were coded as 0.	Continuity Convoy model
Ingersoll-Dayton et al.	1988	Cross-sectional Personal interview	USA	Elders and their friends All members of a sampled household 70 y and older were interviewed	≥50 y (Range 50–95)	*N* = 718	Amount of perceived reciprocity: number who received support and number who provided support Network demand General well-being.	If the functional supports were calculated as zero, this indicated a reciprocal relationship. A positive number were coded as receiving more. A negative number were coded as giving more.	Continuity Life satisfaction Negative affect

Lewittes	1989	Cross-sectional Two studies: (1) Quantitative (2) Qualitative	USA; Long Island and New York City area White and black women	Elderly women and their friends (1) Questionnaire study (2) Interviews of both members of four friendship pairs	≥65 y	(1) *N* = 169 (2) *N* = 8 (four friend-ship pairs)	(1) Quantitative: Emotional support-Intimacy, Activity, Practical help (2) Qualitative: Emotional support-Intimacy, Activity, Practical help	Relationships are measured in terms of the equity of exchange input and exchange outcome and the gains and losses of each person in the relationship	Continuity Making new friends

James et al.	1984	Longitudinal (4 y) 1978–1982 Participants observation Interview	Ireland Three coastal hamlets	Elderly inhabitants and their close family members, remote kin, neighbors, and parental caretakers	≥60 y	*N* = 70	All kinds of social interaction and social support: Emotional support Instrumental support Social companionship	A life history method—collecting detailed qualitative information Interview focusing on particular economic and social variables, that is, questions about the degree of interaction (i.e., reciprocity) with close family, their neighbors, and kin.	Continuity

de Vugt et al.	2003	Longitudinal (2 y) Questionnaire Interview	The Netherlands	Female and male spouse caregivers of consecutively referred patients with dementia	Caregiver: M = 68.3 (SD 7.9) Patient: M = 71.6 (SD 7.6)	*N* = 53 (male = 22; female = 31)	The quality of the relationship was measured by four items: General closeness, Communication, Similarity of views about life, and Degree of getting along. Interviews reporting on changes in their relationship since the onset of dementia	Analysis of the difference between baseline and the followup questionnaires and interviews. Description of relational changes since the onset of dementia	Relationship changes (i.e., reciprocity) Continuity of the relationship
Hooker et al.	2000	Cross-sectional Questionnaire Interview	USA New York	Female and male spouse caregivers for patients with Alzheimer' (AD) and Parkinson' diseases (PD)	Caregivers AD: M = 71 y Caregivers PD: M = 67y	*N* = 175 spouse caregivers AD *N* = 88 (male = 36; female = 52) PD *N* = 87 (male = 32; female = 55)	Coping strategies: Problem-Focused coping Social support coping Emotion-focused coping Depression Anxiety	Analysis of answers from the questionnaires and the interview	Marital satisfaction. Mental health Continuity

Neufeld et al.	1998	Longitudinal (18 months) Three waves In-depth interviews Focus-group discussion	Canada	Male caregivers of cognitively impaired (primarily AD) older adults. Caregivers relationship to care recipient, family, and friends	≥60 y (range 33 to 87 y)	*N* = 22 caregivers	Describing a typical day Describing give-and-take in all their relationships and the support they received from and gave to others	Coding and analysis of the presence or absence of reciprocity, the context in which reciprocity occurred, characteristics of reciprocity, and the consequent feelings of the caregiver were coded, and analyzed	Continuity

Bear	1990	Longitudinal Two waves At entry in an RCH resident and six months after entering an RCH Face-to-face interviews	USA Florida	The elderly and their relations to their family and friends chosen upon emotional bonding and tie content	≥60 y	*N* = 81	Measurement of network density, reciprocity, intensity, and material linkages The year prior to their RCH entry, retrospectively, by their present entry period and six months later	Reciprocity was measured by the proportion of each resident's network material links (financial, assistance (including past assistance from the residents), gifts) that are reciprocated	Continuity

Knesebeck et al.	2003	Cross-sectional Telephone interviews	Germany USA	Elderly and their relationship to their marital life (or partnership) The parent-children exchange	≥60 y	*N* = 1290 Germany *N* = 682 USA *N* = 608	Emotional support Practical support Experience regarding reciprocity/nonreciprocity of their most important social relationships	The effort-reward-model: (effort spent –“high cost” and rewards received –“low gain”)	Mental health Depressive symptoms: CES-D scale
McMunn et al.	2009	Cross-sectional	England	The elderly	M = 62 y	*N* = 5384	“Caring for others” defined as emotional and instrumental social support	The effort-reward model	Mental Health: Quality of life—CASP-scale Life satisfaction—SLS-scale Depressive symptoms—CES-D scale

Wahrendorf et al.	2006	Cross-sectional Face-to face-interviews	Ten European countries (Austria, Germany, Sweden, The Netherlands, Spain, Italy, France, Denmark, Greece, and Switzerland)	The elderly	≥50 y	*N* = 22 000	“Care for a sick or disabled adult” “Provide help to family, friends, or neighbors”	The effort-reword model	Mental health: Quality of life—CASP-scale Depressive symptoms—CES-D scale

Liang et al.	2001	Cross-sectional	USA Different counties	The elderly and their relationships with their friends, neighbors, and relatives	≥65 y	*N* = 1103	Emotional support Tangible support Informational support	Assessed composites measures of both support received and given	Mental health: Depressive symptoms—CES-D scale

Roberto et al.	1986	Cross-Sectional Interview	USA	The elderly and their relationship with their best friend	≥65 y M = 73.8 y	*N* = 110	Emotional support Instrumental support Social companionship	Making an equity score based on a modified version of the Walster Global Measure of Participants' perceptions of Inputs, Outcomes, and Equity/Inequity: Scores less than zero were “overbenefited”. Scores equal to zero were “equitable benefited”. Scores greater than zero were “underbenefited”.	Mental health: Relationship Distress—“Austin's Total Mood Index”
Rook	1987	Cross-sectional	USA Los Angeles	Elderly widowed women and their relationships with their social network, in particular with friends and family members	≥60 y M = 72 y	*N* = 120	Emotional support Instrumental support Social companionship	Numbers of positive inputs received and positive inputs provided were computed as two measures. A difference score was computed by subtracting these two measures: 0 represented an “equitable exchange pattern”; a positive score indicated an “overbenefited” position; and a negative score indicated an “underbenefited” position.	Mental health/Social satisfaction: Loneliness—UCLA and SFL scales Psychological well-being (Bradburn, 1969) Quality of life (Campbell et al., 1976)

Nemoto	1998	Cross-Sectional Telephone Interview and questionnaires	USA	Japanese American elderly resided in New York	≥55 y M = 71 y	*N* = 50	Emotional support Instrumental support	Reciprocity norms were identified by asking respondents to rate their perception of the reciprocal behaviors in each function of social support on a 7-point response scale	Mental health: Life satisfaction (Rapkin et al., 1992) Scale of depressive symptoms (*α* = .80)

Wentowski	1981	Anthropological study Structured interviews Participant observation	USA Cities from the industrial complexes in southern USA	The elderly and their exchanges to persons in different roles in their social network	≥55 y M = 71 y	*N* = 50	Identify exchange strategies within the personal networks of transference of goods services, emotional support and the cultural rules of reciprocity directing these exchanges.	Identifying the cultural rules governing reciprocity as the basis for constructing exchange strategies	Mental health: Degree of independence Degree of self-worth
